# Risk Assessment of Dyslipidemias, Hyperglycemia, Hyperuricemia, and Hypertension Utilizing Self-Reported Body Silhouettes

**DOI:** 10.1155/2023/4991684

**Published:** 2023-03-28

**Authors:** Ruben Blachman-Braun, Juan O. Talavera, Marcela Pérez-Rodríguez, Ivonne Roy-García, Rodolfo Rivas-Ruiz, Gerardo Huitrón-Bravo, Jorge Salmerón

**Affiliations:** ^1^Department of Urology, University of Miami Miller School of Medicine, Miami, FL, USA; ^2^Training and Clinical Research Center, Specialty Hospital, Mexican Social Security Institute, National Medical Center XXI, Mexico City, Mexico; ^3^Teaching Headquarters, ABC Medical Center, Mexico City, Mexico; ^4^Faculty of Medicine, Autonomous University of the State of Mexico, Toluca, State of Mexico, Mexico; ^5^Center for Research in Policies, Population and Health, National Autonomous University of Mexico, Mexico City, Mexico

## Abstract

**Objective:**

Self-reported body silhouette is an anthropometric instrument that has been utilized as a screening tool for underweight, overweight, obesity, and other abnormal anthropometric variables. Herein, we analyzed the risk associated with the self-reported body silhouette in the scope of dyslipidemias, hyperglycemia, hyperuricemia, and hypertension.

**Methods:**

Adult participants of the Health Workers Cohort Study enrolled between March 2004 and April 2006 were included. Then, risk analysis was performed considering dyslipidemias as serum triglycerides, high total cholesterol, high LDL-C, low HDL-C, hyperglycemia, hyperuricemia, and hypertension.

**Results:**

A total of 2,297 males and 5,003 females were analyzed. The median ages of the studied population was 39 (30–49) and 41 (31–50) years for males and females, respectively. Overall, there is a stepwise increase in the risk of presenting dyslipidemias, hyperglycemia, hyperuricemia, and hypertension as the self-reported body silhouette number increases, this tendency was observed in both males and females.

**Conclusion:**

Self-reported body silhouette is a useful risk assessment tool for dyslipidemias, hyperglycemia, hyperuricemia, and hypertension in Mexican adults. Applications of questioners containing this silhouette might be considered a valuable public health instrument due to their low cost, relative simplicity, and absence of specialized equipment, training, or respondent knowledge.

## 1. Introduction

Cardiovascular disease (CVD) is one of the most prevalent public health problems worldwide. Due to the high morbidity and mortality associated with this group of pathologies [1], in recent decades, we have developed a better understanding of the clinical and metabolic factors associated with CVD such as hypertension [2], dyslipidemias [3, 4], hyperglycemia, and hyperuricemia [5]. Thus, in order to modify the cardiovascular risk, it has become a common practice that physicians order tests to assess those variables. However, the quantification of biochemical and clinical parameters requires special instruments, trained personnel, and resources that are in some clinical settings not always available.

Self-reported body silhouette is an anthropometric tool that was initially postulated by Sorensen et al. [6], and since then it has been validated and modified by several authors [7–10]. We have shown that this instrument is useful as a screening tool for overweight, obesity, increased waist circumference, and elevated body fat percentage [11]. Furthermore, some studies have indicated that there is a correlation between anthropometric parameters such as body mass index (BMI) and serum lipids parameters [12], uric acid, glucose, and blood pressure [13]. However, it is not always feasible to take measurements of height, weight, and BMI in large-scale epidemiological studies, so the proposal of this study is to evaluate body image as a tool to identify those subjects with higher metabolic risk and analyze the association between self-reported body silhouette and dyslipidemias, hyperglycemia, hyperuricemia, and hypertension. We hypothesize that self-reported body silhouette is a useful risk assessment tool for dyslipidemias, hyperglycemia, hyperuricemia, and hypertension in Mexican adults.

## 2. Materials and Methods

### 2.1. Participants Selection

Adults from the Health Workers Cohort Study were analyzed in this study. This cohort included medical, administrative, and academic employees (and their families), that were at the time of the evaluation working either at the Mexican Social Security Institute (in Cuernavaca, Morelos), the National Institute of Public Health (in Cuernavaca, Morelos), or the Autonomous University of Mexico State (in Toluca, State of Mexico) [14].

All adult participants enroll between March 2004 and April 2006 were assessed for eligibility, then we excluded those with a past medical history of anorexia or bulimia, clinical scenarios that are associated with edema (i.e., chronic kidney disease, liver cirrhosis, myocardial infarction, angina, history of heart surgery), cerebrovascular disease, diabetes mellitus, and those who underwent a weight reduction program within the last 6 months. Additionally, we further excluded those participants under medical treatment for heart failure or on medication that affects the lipid panel (i.e., beta-blockers, diuretics, and anticholesterolemic) and those without the self-reported body silhouette.

### 2.2. Clinical Variables and Self-Reported Body Silhouette Assessment

**Figure 1:**
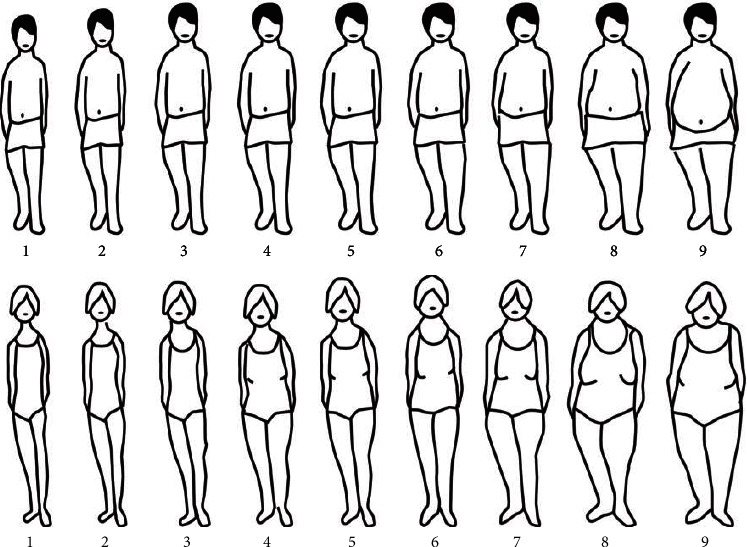
Male and female drawings of the body silhouette.

The past medical history, clinical, and demographic characteristics were recorded through self-administered questionnaires [15]. In those, the participants were asked to identify their current body silhouette on a scale from 1 to 9, with 1 being the thinnest silhouette and 9 being the silhouette of someone with morbid obesity. The body silhouettes are composed of two different sets, one for males and the other for females. The silhouette used in this research were those initially postulated by Sorensen et al. [6] (Figure 1). Additionally, the participant's civil or marital status and the highest level of educational attendance were assessed. For the analysis of the educational level, patients were classified as either Primary school (in the Mexican education system: Escuela Primaria), middle school (in the Mexican education system: Escuela Secundaria), high school (in the Mexican education system: Escuela Preparatoria or Normal Superior), or those planning to attend graduate school or postgraduate school.

Bodyweight was measured with a calibrated electronic scale (model BC-533; Tanita, Tokyo, Japan) with participants wearing minimum clothing and without shoes and socks. Height was measured using a conventional stadiometer (SECA brand), with barefoot participants standing with their shoulders in a normal position; the measurements were taken with a measuring tape in a horizontal plane perpendicular to the vertical scale, touching the top of the head at the moment of inspiration. Then BMI was calculated with the standard formula (weight in kg/(height in m)^2^).

### 2.3. Biochemical Analysis

Paraclinical tests were carried out using a venous blood sample taken after 8 hours or longer fasting; approximately 20 ml of venous blood was obtained from each participant. Then, plasma triglycerides were measured with a colorimetric method following enzymatic hydrolysis performed with the lipase technique. Furthermore, the total cholesterol was measured by the colorimetric method following an enzymatic assay. Additionally, low-density lipoprotein cholesterol (LDL-C) and high-density lipoprotein cholesterol (HDL-C) were measured with the clearance method. In addition, serum glucose and uric acid concentration were obtained. All biomedical assays were performed using a Selectra XL instrument (Randox), in accordance with the International Federation of Clinical Chemistry and Laboratory Medicine Guidelines [16].

### 2.4. Lipids, Glucose, Uric Acid, and Blood Pressure Profile Assessment

Dyslipidemias were defined according to the criteria set by the National Cholesterol Education Program ATP-III, which defines a high lipid profile as high serum triglycerides ≥150 mg/dL, high serum total cholesterol ≥200 mg/dL, high LDL-C ≥100 mg/dL, and low HDL-C <40 mg/dL for males and <50 mg/dL for females [17]. Hyperglycemia was considered as a serum glucose concentration of ≥100 mg/dL, per the American Diabetes Association standards for prediabetes [18]. Additionally, uric acid above ≥7 mg/dl was considered hyperuricemia [19].

During the participants' visit, blood pressure was measured twice by a trained nurse using an automatic monitor. The first measurement was performed after the patient rested for 5 minutes, while the participant was sitting with the dominant arm supported at the level of the heart. The second measurement was taken using the same methodology five minutes apart from the first blood pressure measurement, and the mean of both recordings was used as a variable for the statistical analysis. The nurses who performed all measurements were trained in the use of standardized procedures (reproducibility was evaluated, resulting in concordance coefficients between 0.83 and 0.90) [20]. Furthermore, hypertension was considered to have a diastolic blood pressure ≥90 mmHg and a systolic blood pressure ≥140 mmHg [21]. For the purpose of the statistical analysis, diastolic and systolic blood pressures were analyzed as a single variable each.

### 2.5. Statistical Analysis

The statistical analysis was performed with the SPSS version 24 program for Windows. Continue variables were presented with either the mean and standard deviation (±SD) or median and interquartile range (25^th^–75^th^), depending on the data distribution. Categorical variables were presented as absolute values and frequencies. Differences in biochemical markers were compared according to the type of body silhouette, a Spearman's correlation was performed. Additionally, for the risk analysis, the second self-reported body silhouette in both males and females, was considered the reference value (Odds ratio (OR) = 1). Furthermore, the *β* and standard error values were obtained through the binary logistical regression analysis adjusted for age, then those values were transferred to the RevMan 5.3 program (Cochrane Collaboration) to create the OR plots. For the purpose of this study a *p* value <0.05 was considered statistically significant.

## 3. Results

**Figure 2:**
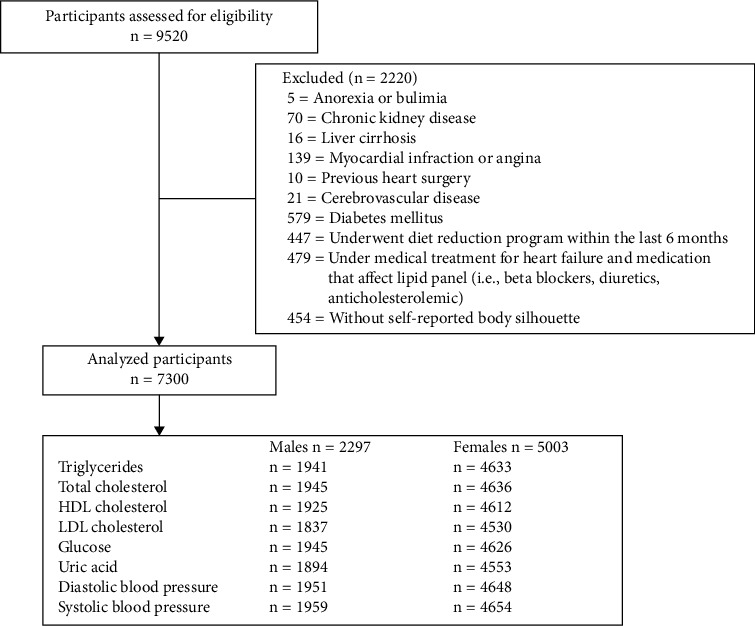
Participants' selection.

After excluding 2,220 participants, a total of 2,297 males and 5,003 females were analyzed, and for each clinical variable, the exact number of patients analyzed is presented in Figure 2. The median age of the studied population was 39 (30–49) and 41 (31–50) years for males and females, respectively, and the BMI was 26.59 ± 4.03 and 26.08 ± 4.5 kg/m^2^ for males and females, respectively. The rest of the clinical and demographic characteristics, in addition to the frequency of increased blood pressure, dyslipidemias, hyperglycemia, and hyperuricemia, are present in Table 1.

The medians of the biochemical markers, BMI, and blood pressure in accordance with self-reported body silhouette and sex are shown in Table 2. Correlational analysis was statistically significant (*p* < 0.001) for all variables; the correlation was a positive correlation for all markers, except for HDL, which shows a negative correlation.

**Figure 3:**
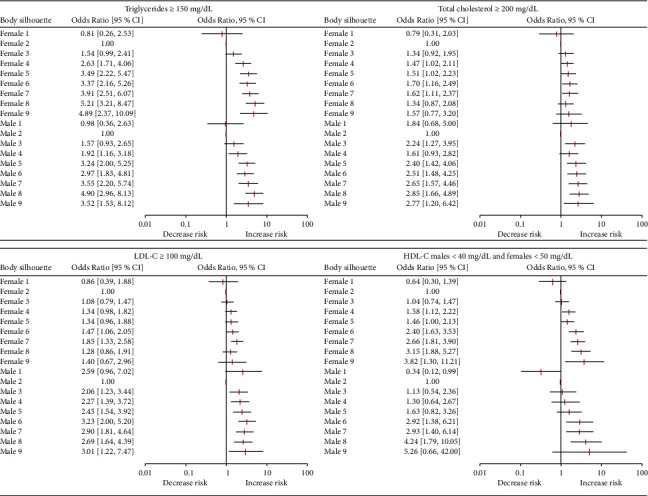
Risk assessment of triglycerides ≥150 mg/dL, total cholesterol ≥200 mg/dL, LDL-C ≥100 mg/dL, and low HDL-C (males <40 mg/dL, females <50 mg/dL), for each self-reported body silhouette for both females and males. All models were adjusted by age.

The risk analysis indicated that in both males and females, there is a tendency toward an abnormal lipid profile that increased with a higher self-reported body silhouette. Thus, the risk for triglycerides ≥150 mg/dL in females was statistically significant from silhouette 4 (OR = 2.63, 95% confidence interval (CI): 7.71–4.06) through silhouette 9 (OR = 4.89, 95% CI: 2.37–10.09); for males, the risk was also significant from silhouette 4 (OR = 1.92, 95% CI: 1.83–4.81) through 9 (OR = 3.52, 95% CI: 1.53–8.12). Although in females the risk of total cholesterol ≥200 mg/dL was only statistically significant on silhouettes 6 (OR = 1.70, 95% CI: 1.16–2.49) and 7 (OR = 1.62, 95% CI: 1.11–2.37), in males it was significant from silhouette 3 (OR = 2.24, 95% CI: 1.27–3.95) through 9 (OR = 2.77, 95% CI: 1.20–6.42). Though in females, LDL-C ≥100 mg/dL appears to increase in a stepwise pattern with each silhouette, only silhouette 7 is statistically significant (OR = 1.85, 95% CI: 1.33–2.58), in contrast for males there was a significant increase from silhouette 3 (OR = 2.06, 95% CI: 1.23–3.44) through 9 (OR = 3.01, 95% CI: 1.22–7.47). Additionally, females were at risk from HDL-C <50 mg/dL from silhouette 4 (OR = 1.58, 95% CI: 1.12–2.22) through 9 (OR = 3.82, 95% CI: 1.30–11.21), and males were at risk of HDL-C <40 mg/dL from silhouette 6 (OR = 2.92, 95% CI: 1.38–6.21) through 8 (OR = 4.24, 95% CI: 1.79–10.05) (Figure 3).

**Figure 4:**
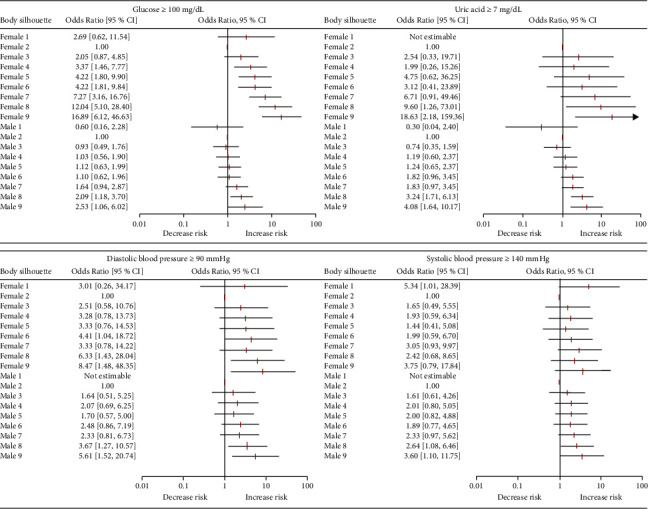
Risk assessment of glucose ≥100 mg/dL, uric acid ≥7 mg/dL, diastolic blood pressure ≥90 mmHg, and systolic blood pressure ≥140 mmHg, for each self-reported body silhouette for both females and males. All models were adjusted by age.

It was observed that the risk of hyperglycemia (glucose ≥100 mg/dL) was significantly increased in females from silhouette 4 (OR = 3.37, 95% CI: 1.47–7.77) through 9 (OR = 16.89, 95% CI: 6.12–46.63), and for males in silhouettes 8 (OR = 2.09, 95% CI: 1.18–3.70) and 9 (OR = 2.53, 95% CI: 1.06–6.02). In relation to hyperuricemia (uric acid ≥7 mg/dL), for females silhouettes 8 (OR = 9.60, 95% CI: 1.26–73.01) and 9 (OR = 18.63, 95% CI: 2.18–159.36), and for males silhouettes 8 (OR = 3.24, 95% CI: 1.71–6.13) and 9 (OR = 4.08, 95% CI: 1.64–10.17) were statistically significant (Figure 4).

After calculating the risk for hypertension, it was shown that diastolic blood pressure ≥90 mmHg was significantly increased in females in silhouettes 8 (OR = 6.33, 95% CI: 1.43–28.04) and 9 (OR = 8.47, 95% CI: 1.48–48.35); in males also silhouettes 8 (OR = 3.67, 95% CI: 1.27–10.57) and 9 (OR = 5.61, 95% CI: 1.52–20.74) had a statistically significant risk. For systolic blood pressure ≥140 mmHg, though in females it seems that there is an increase in the risk with each silhouette, the only silhouette with a statistically significant risk was 1 (OR = 5.34, 95% CI: 1.01–28.39); for males silhouettes 8 (OR = 2.64, 95% CI: 1.08–6.46) and 9 (OR = 3.60, 95% CI: 1.10–11.75) were statistically significant (Figure 4).

## 4. Discussion

Our results indicate that the self-reported body silhouette is a useful risk assessment tool for dyslipidemias, hyperglycemia, hyperuricemia, and hypertension in Mexican adults. We find that there is a stepwise increase in the risk of presenting these abnormal parameters as the self-reported body silhouette number increased, this tendency was observed in both males and females.

The underlying pathophysiology that supports our observations might be attributed to the intrinsic correlation of the self-reported body silhouettes and anthropometric parameters (i.e., BMI and body fat percentage). This observation is supported by a previous study performed on the Mexican population [22], in which a strong correlation between the BMI and the Stunkard's silhouette (males: *r* = 0.702, females: *r* = 0.766) was reported. In addition, using another type of silhouettes, the Pulver's silhouettes, Yepes et al. [23] also found a strong correlation between reported body silhouettes and BMI (males: *r* = 0.80, females: *r* = 0.81) and body fat percentage (males: *r* = 0.71, females: *r* = 0.73) within an African cohort. Thus, as the BMI increase so does the risk of pathological conditions (i.e., dyslipidemia, diabetes mellitus, hyperuricemia, and hypertension) becomes higher [12, 13, 24]. Hence, this opens the possibility of using the self-reported body silhouettes as a screening tool to indirectly assess the risk of those pathological states, particularly in rural areas in which special instruments and trained healthcare professionals are not always available. In our cohort, we found that there is a positive correlation between self-reported body silhouettes and biochemical markers, such as BMI and blood pressure, except for a negative correlation between HDL self-reported body silhouettes.

In the present study, the association with dyslipidemias remains strong with the higher self-reported body silhouette (silhouettes: 6, 7, 8, and 9); this association persists after correcting the analysis by age, a variable that strongly influences the lipid profile in healthy patients [25]. Our findings might be attributed to the proxy measurement of adiposity performed by the self-reported body silhouette, as it has been reported that excessive body fat percentage is a main metabolic comorbidity associated with dyslipidemia. [26] Although there is no substantial change in the overall risk of dyslipidemias among silhouettes 6 through 9, it is clear that there is a strong tendency for the higher silhouette to have dyslipidemia; nonetheless, in some silhouette, the CI 95% suggests that the association is statistically significant (i.e., females with silhouettes 8 and 9 associated with high LDL-C, and males on silhouette 9 with low HDL-C) this is probably due to the small number of participants analyzed within those particular body silhouettes.

Current evidence strongly supports the notion that hyperglycemia is a cardiovascular risk factor [27]. Moreover, it has been reported that an increased visceral abdominal fat is associated with insulin resistance (OR = 1.77, 95% CI: 1.04–3.02) [28]. Hence, with an increase in the visceral abdominal fat with each silhouette's number, there is a higher risk of hyperglycemia (particularly in females) with higher self-reported body silhouettes.

Uric acid seems to have antioxidant activity in the extracellular environment; however, once it enters the intracellular environment (i.e., vascular smooth muscle cells and adipocytes), this molecule has harmful effects, which include the inhibitory effect on nitric oxide production, the induction of platelet aggregation, and pro-inflammatory activity. Thus, it has been proposed that hyperuricemia could independently increase the risk of the metabolic syndrome, diabetes mellitus, hypertension, renal disease, gout, and CVD [29], mainly in patients with overweight and obesity [30]. Moreover, anthropometric variables such as the BMI have been significantly correlated with serum uric acid levels (male: *r* = 0.235, female: *r* = 0.140) [31]. Hence, it is possible that the intrinsic correlation of the BMI and the self-reported body silhouettes is responsible for the stepwise increase in the risk of hyperuricemia observed in both male and female silhouettes.

A study performed in the Framingham cohort reported that the interarm systolic blood pressure difference ≥10 mm Hg was significantly associated with an increased incidence of cardiovascular events (hazard ratio = 1.38, 95% CI: 1.09–1.75) [32]. Furthermore, it has been shown that a reduction in systolic blood pressure decreases the risk of CVD and all-cause mortality [33]. Thus, from a clinical standpoint, it is of great interest to monitor blood pressure and diagnose those with hypertension. Although self-reported body silhouettes cannot measure blood pressure parameters, in our research, we have shown that self-reported body silhouettes can assess the risk of diastolic and systolic hypertension, mainly in silhouettes 8 and 9. The strong association seen in this analysis might be adjudicated to the intrinsic relation between the body silhouettes and anthropometric parameters (i.e., BMI and body fat percentage), as Dua et al.'s [34] study indicated that an increase in BMI correlates with increased diastolic (male: *r* = 0.32; female: *r* = 0.35) and systolic (male: *r* = 0.26; female: *r* = 0.30) blood pressure. This correlation is also significant when analyzing body fat percentage and diastolic (male: *r* = 0.51; female: *r* = 0.42) and systolic (male: *r* = 0.42; female: *r* = 0.47) blood pressure.

There are several limitations of utilizing self-reported body silhouettes as a clinical instrument, this due to the test-taking ability and body perception that may change according to the socioeconomic status, illiteracy, and educational level of each individual [35]. Additionally, as has been indicated by Osuna-Ramírez et al. [10], a limitation of this study is that our cohort has a high percentage of health-related workers and overall a higher degree of education compared to the average Mexican population; for that reason, our results may not represent the complete picture of the general Mexican population.

Despite the limitations of this study, our findings provide an important insight into the application of self-reported Sorensen's body silhouette as a simple risk assessment instrument. Given their relative simplicity in the administration of those questioners and the absence of specialized equipment, training, or respondent knowledge, the self-reported body silhouette might be considered a low-cost screening epidemiological instrument [23], useful in the screening of individuals at risk of dyslipidemias, hyperglycemia, hyperuricemia, and hypertension. Additionally, the self-reported body silhouette may also be utilized in public health promotion mediatic campaigns, in which interactive methodological strategies that ask individuals to identify themselves within the body silhouette scale, provide automatic feedback to each participant on the cardiovascular risk factors. Consequently, this public health intervention will indicate participants with a higher risk of CVD to initiate lifestyle changes and will refer them to their primary care physician. Though the findings in our research are encouraging, further studies that determine the sensibility, specificity, and positive likelihood ratio to each self-reported body silhouette in other educational strata, might help clinicians better determine the utility of the self-reported body silhouettes as a screening instrument for dyslipidemias, hyperglycemia, hyperuricemia, and hypertension. It is our opinion that a self-reported body silhouette will not replace the standardized and well-validated anthropometric screening tools but will assist as an additional instrument in the arsenal that medical providers have to improve the detection of cardiovascular risk factors in the overall population.

## 5. Conclusion

Self-reported body silhouette is a useful risk assessment tool for dyslipidemias, hyperglycemia, hyperuricemia, and hypertension in Mexican adults. Applications of questioners containing this silhouette might be considered a valuable public health instrument due to their low cost, relative simplicity, and absence of specialized equipment, training, or respondent knowledge.

## Figures and Tables

**Table 1:** Clinical and demographic characteristics of the studied patients.

	Males *n* = 2297 (%)	Females *n* = 5003 (%)
Age (years)	39 [30–49]	40 [31–49]
Civil status		
Married/free union	1811 (78.8)	2910 (58.2)
Divorced/separated	85 (3.7)	532 (10.6)
Widower/widow	24 (1)	304 (6.1)
Single	353 (15.4)	1195 (23.9)
Not reported	24 (1)	62 (1.2)
Highest level of educational attendance		
Primary school	145 (6.3)	478 (9.6)
Middle school	264 (11.5)	630 (12.6)
High school	377 (16.4)	941 (18.8)
Graduate school/postgraduate school	1148 (50)	2208 (44.1)
Not reported	363 (15.8)	746 (14.9)
Self-reported body silhouette		
1	26 (1.1)	36 (0.7)
2	117 (5.1)	238 (4.8)
3	220 (9.6)	1027 (20.5)
4	291 (12.7)	1239 (24.8)
5	422 (18.4)	587 (11.7)
6	411 (17.9)	707 (14.1)
7	460 (20)	852 (17)
8	315 (13.7)	269 (5.4)
9	35 (1.5)	48 (1)
Weight (kg)	76 ± 12.95	63.28 ± 11.44
Height (cm)	168.95 ± 6.94	155.80 ± 6.25
BMI (kg/m^2^)	26.59 ± 4.03	26.08 ± 4.50
Triglycerides (mg/dL)	164 [112.5–234]	120 [85–167.5]
Triglycerides ≥150 mg/dL	1087 (47.3)	1487 (29.7)
Total cholesterol (mg/dL)	191.4 [167–218.6]	186 [162.9–213.3]
Total cholesterol ≥200 mg/dL	796 (34.7)	1711 (34.2)
LDL cholesterol (mg/dL)	116 [91–138]	114 [89–139]
LDL-C ≥100 mg/dL	1249 (54.4)	2940 (58.8)
HDL cholesterol (mg/dL)	37 [32–42]	39 [33–47]
Low HDL-C (males <40 mg/dL, females <50 mg/dL)	1778 (77.4)	3790 (75.8)
Glucose (mg/dL)	92 [85–100]	87 [81–94]
Hyperglycemia (≥100 mg/dL)	487 (21.2)	655 (13.1)
Uric acid (mg/dL)	5.8 [4.97–6.7]	4.25 [3.46–5.1]
Hyperuricemia (≥7 mg/dL)	396 (17.2)	129 (2.6)
Diastolic blood pressure (mmHg)	74 [67.3–82]	70 [63–77]
Diastolic hypertension (≥90 mmHg)	180 (7.8)	160 (3.2)
Systolic blood pressure (mmHg)	123 [115–132]	112 [106–121]
Systolic hypertension (≥140 mmHg)	239 (10.4)	178 (3.6)

Mean ± standard deviation, median (interquartile range 25^th^–75^th^).

**Table 2:** Biochemical or anthropometric markers according to the type of body silhouette.

	Biochemical or anthropometric markers	Self-reported body silhouette									Rho S	*p*
		1	2	3	4	5	6	7	8	9		
Male	BMI (kg/m^2^)	20.5 [19.2–22.7]	22.5 [20.6–24.1]	23.5 [21.8–25.4]	24.1 [22.7–26.3]	25.4 [23.8–27.2]	26.5 [24.7–28.3]	27.9 [26.2–29.8]	30.4 [28.4–33.1]	34.7 [29.4–42.3]	0.635	<0.001
	Triglycerides (mg/dL)	114 [87–154]	111 [83–175]	125.5 [87.3–201.8]	143 [93–200.5]	172.5 [115.3–256.5]	169 [115–225.5]	175 [125–257.3]	195 [138–270]	183 [123–255]	0.241	<0.001
	Total cholesterol (mg/dL)	176.8 [152–210]	173 [155.7–196.8]	185.5 [159–212.9]	184 [160.5–209 0.7]	192.6 [167–217]	194 [170.8–218.2]	196 [173–222.1]	198.4 [171.5–226.8]	189 [163.5–239.9]	0.138	<0.001
	LDL cholesterol (mg/dL)	105 [84–122.5]	95 [80–121]	109.5 [86–139.8]	114 [88–134]	115 [93–136]	122 [96.8–142]	119 [93–141]	118 [94–141.3]	127 [96–149]	0.113	<0.001
	HDL cholesterol (mg/dL)	44.5 [35.5–51]	40 [33–46]	39 [33–45]	38 [33–42]	37 [31.5–43]	37 [31–42]	36 [32–41]	36 [30.3–40]	36 [28.5–40.5]	−0.140	<0.001
	Glucose (mg/dL)	86 [80–92]	89 [81–95]	89 [84–96]	90 [83.5–97]	91 [85–98]	91 [85–98]	36 [87–102]	36 [89–104]	97 [89–106.5]	0.210	<0.001
	Uric acid (mg/dL)	5.2 [3.8–6.1]	5.3 [4.6–6.3]	5.4 [4.6–6.2]	5.7 [4.8–6.5]	5.7 [4.9–6.5]	5.9 [5.1–6.8]	5.9 [5.1–6.8]	6.3 [5.4–7.3]	6.4 [5.6–7.8]	0.210	<0.001
	Diastolic blood pressure (mmHg)	71 [64–76.8]	70 [63–75]	73 [67–80]	73 [67.7–80.7]	73 [67–81]	75 [67–82]	76 [68.9–83]	76 [69–84]	81 [70–86.7]	0.140	<0.001
	Systolic blood pressure (mmHg)	71 [112.3–123.8]	116 [108–127]	121 [112–130.8]	121.2 [114–131.3]	122 [115–130]	123 [114.5–132]	[116.2 124 133]	126 [119–135.6]	132 [125–138]	0.167	<0.001

Female	BMI (kg/m^2^)	19.4 [18.2–20.5]	20.5 [19.2–21.7]	22.4 [20.9–23.7]	25 [23.4–26.6]	25.8 [23.9–27.9]	26.7 [24.9–28.6]	29.7 [27.6–32]	33.1 [30.6–35.5]	37.9 [34.5–42.2]	0.742	<0.001
	Triglycerides (mg/dL)	85 [61–128.5]	84 [66–121]	96 [73–136]	116 [85–160]	125 [87–174]	129 [93.8–182]	140 [106–189]	152 [114–203.3]	150 [114.8–183.3]	0.299	<0.001
	Total cholesterol (mg/dL)	184.3 [150.7–200.4]	173.2 [154.7–194.6]	177.1 [155–204.9]	184 [163–208.6]	185.3 [165.4–219]	191 [168.2–220]	195.3 [172.4–223]	193.4 [169–214.7]	197.3 [166.8–223.3]	0.172	<0.001
	LDL cholesterol (mg/dL)	101 [67–127.5]	99.5 [78.3–124.5]	105 [81–131]	111 [89–136]	113 [88–142]	120 [93–144.8]	124 [100–148]	121 [96–143]	126 [94–148.5]	0.181	<0.001
	HDL cholesterol (mg/dL)	41 [34.8–59.3]	45 [36–50]	42 [34–50]	39 [33–47]	41 [34–47]	38 [32–45]	37 [32–44]	37 [31–43]	39 [34.8–45.3]	−0.153	<0.001
	Glucose (mg/dL)	81.5 [75.8–89.3]	83 [77–89]	84 [79–90]	86 [81–92]	87 [80.3–93]	88 [82–95]	91 [85–99]	96 [88–103]	93 [85.8–108]	0.298	<0.001
	Uric acid (mg/dL)	3.8 [3.2–4.3]	3.6 [2.9–4.3]	3.9 [3.1–4.6]	4.1 [3.4–4.9]	3.4 [4.2–5.1]	4.5 [3.7–5.2]	4.7 [3.9–5.5]	5 [4.3–6]	5 [4.1–6.3]	0.303	<0.001
	Diastolic blood pressure (mmHg)	69 [60–70.8]	68 [60–74]	68 [61–74]	69.7 [62–75.9]	71 [65–78]	70 [64–77.7]	73 [66–79]	74 [68–80]	76 [68–82.5]	0.210	<0.001
	Systolic blood pressure (mmHg)	110 [104.5–119]	110 [102.3–116.8]	109.5 [103–115]	110 [105–120]	113 [106–121.7]	113 [106–121.3]	117 [110–126]	119 [110–129]	120 [111.5–131]	0.254	<0.001

Median (interquartile range 25^th^–75^th^); Rho S, rho spearman.

## Data Availability

Data used to support the findings of this study are available upon reasonable request through a data access committee.

## References

[1] Chen Y., Freedman N. D., Albert P. S. (2019). Association of cardiovascular disease with premature mortality in the United States. *JAMA Cardiology*.

[2] Van Oort S., Beulens J. W. J., Van Ballegooijen A. J., Grobbee D. E., Larsson S. C. (2020). Association of cardiovascular risk factors and lifestyle behaviors with hypertension: a mendelian randomization study. *Hypertension*.

[3] Atar D., Jukema J. W., Molemans B. (2021). New cardiovascular prevention guidelines: how to optimally manage dyslipidaemia and cardiovascular risk in 2021 in patients needing secondary prevention?. *Atherosclerosis*.

[4] Sandesara P. B., Virani S. S., Fazio S., Shapiro M. D. (2019). The forgotten lipids: triglycerides, remnant cholesterol, and atherosclerotic cardiovascular disease risk. *Endocrine Reviews*.

[5] Johnson R. J., Bakris G. L., Borghi C. (2018). Hyperuricemia, acute and chronic kidney disease, hypertension, and cardiovascular disease: report of a scientific workshop organized by the national kidney foundation. *American Journal of Kidney Diseases*.

[6] Sørensen T. I., Stunkard A. J., Teasdale T. W., Higgins M. W. (1983). The accuracy of reports of weight: children’s recall of their parents’ weights 15 years earlier. *International Journal of Obesity*.

[7] Anjos L. A. D., Moraes C. F. (2020). Agreement between self-assessment of body image and measured body mass index in the Brazilian adult population. *Ciência and Saúde Coletiva*.

[8] Keirns N. G., Hawkins M. A. W. (2019). The relationship between intuitive eating and body image is moderated by measured body mass index. *Eating Behaviors*.

[9] Toselli S., Grigoletto A., Zaccagni L. (2021). Body image perception and body composition in early adolescents: a longitudinal study of an Italian cohort. *BMC Public Health*.

[10] Osuna-Ramírez I., Hernández-Prado B., Campuzano J. C., Salmerón J. (2006). Indice de masa corporal y percepción de la imagen corporal en una población adulta mexicana: la precisión del autorreporte. *Salud Pública de México*.

[11] Blachman-Braun R., Talavera J. O., Pérez-Rodríguez M. (2021). Self-reported body silhouettes: a diagnostic instrument for anthropometric parameters. *Public Health*.

[12] Hertelyova Z., Salaj R., Chmelarova A., Dombrovsky P., Dvorakova M. C., Kruzliak P. (2016). The association between lipid parameters and obesity in university students. *Journal of Endocrinological Investigation*.

[13] Zhang L., Li J. L., Zhang L. L., Guo L. L., Li H., Li D. (2020). Body mass index and serum uric acid level: individual and combined effects on blood pressure in middle-aged and older individuals in China. *Medicine (Baltimore)*.

[14] Denova-Gutiérrez E., Flores Y. N., Gallegos-Carrillo K. (2016). Health workers cohort study: methods and study design. *Salud Pública de México*.

[15] Denova-Gutiérrez E., Flores Y. N., Gallegos-Carrillo K., Ramírez-Palacios P., Rivera-Paredez B., Muñoz-Aguirre P. (2016). Self-administered Questionnaires for the Health Workers Cohort Study. *Salud pública Méx*.

[16] Tate J. R., Berg K., Couderc R. (1999). International federation of clinical Chemistry and laboratory medicine (IFCC) standardization project for the measurement of lipoprotein(a). Phase 2: selection and properties of a proposed secondary reference material for lipoprotein(a). *Clinical Chemistry and Laboratory Medicine (CCLM)*.

[17] Grundy S. M., Stone N. J., Bailey A. L. (2019). 2018 AHA/ACC/AACVPR/AAPA/ABC/ACPM/ADA/AGS/APhA/ASPC/NLA/PCNA guideline on the management of blood cholesterol: a report of the American college of cardiology/American heart association Task Force on Clinical practice guidelines. *Journal of the American College of Cardiology*.

[18] American Diabetes Association (2021). Classification and diagnosis of diabetes: standards of medical care in diabetes-2021. *Diabetes Care*.

[19] FitzGerald J. D., Dalbeth N., Mikuls T. (2020). 2020 American college of rheumatology guideline for the management of gout. *Arthritis Care and Research*.

[20] Huitrón-Bravo G. G., Denova-Gutiérrez E., de Jesús Garduño-García J., Talavera J. O., Herreros B., Salmerón J. (2015). Dietary magnesium intake and risk of hypertension in a Mexican adult population: a cohort study. *BMC Nutrition*.

[21] Bangalore S., Gong Y., Cooper-DeHoff R. M., Pepine C. J., Messerli F. H. (2014). Eighth Joint National Committee panel recommendation for blood pressure targets revisited: results from the INVEST study. *Journal of the American College of Cardiology*.

[22] Kaufer-Horwitz M., Martínez J., Goti-Rodríguez L. M., Ávila-Rosas H. (2006). Association between measured BMI and self-perceived body size in Mexican adults. *Annals of Human Biology*.

[23] Yepes M., Viswanathan B., Bovet P., Maurer J. (2015). Validity of silhouette showcards as a measure of body size and obesity in a population in the African region: a practical research tool for general-purpose surveys. *Population Health Metrics*.

[24] Masocha V., Monyeki M. A., Czyż S. H. (2020). Longitudinal relationships between changes in body composition and changes in selected metabolic risk factors (abdominal obesity and blood pressure) among South African adolescents. *PeerJ*.

[25] Okui T. (2021). An age-period-cohort analysis of prevalence and consultation rate for dyslipidemia in Japan. *Asia-Pacific Journal of Public Health*.

[26] Zhang T., Chen J., Tang X., Luo Q., Xu D., Yu B. (2019). Interaction between adipocytes and high-density lipoprotein:new insights into the mechanism of obesity-induced dyslipidemia and atherosclerosis. *Lipids in Health and Disease*.

[27] Li M., Cui Z., Meng S. (2020). Associations between dietary glycemic index and glycemic load values and cardiometabolic risk factors in adults: findings from the China health and nutrition survey. *Nutrients*.

[28] McLaughlin T., Lamendola C., Liu A., Abbasi F. (2011). Preferential fat deposition in subcutaneous versus visceral depots is associated with insulin sensitivity. *Journal of Clinical Endocrinology and Metabolism*.

[29] Saito H., Tanaka K., Iwasaki T. (2021). Xanthine oxidase inhibitors are associated with reduced risk of cardiovascular disease. *Scientific Reports*.

[30] Sebekova K., Gurecka R., Podracka L. (2020). Asymptomatic hyperuricemia associates with cardiometabolic risk indicators in overweight/obese but not in lean adolescents. *Diabetes, Metabolic Syndrome and Obesity: Targets and Therapy*.

[31] Yue J.-R., Huang C.-Q., Dong B.-R. (2012). Association of serum uric acid with body mass index among long-lived Chinese. *Experimental Gerontology*.

[32] Weinberg I., Gona P., O’Donnell C. J., Jaff M. R., Murabito J. M. (2014). The systolic blood pressure difference between arms and cardiovascular disease in the Framingham heart study. *The American Journal of Medicine*.

[33] Bundy J. D., Li C., Stuchlik P. (2017). Systolic blood pressure reduction and risk of cardiovascular disease and mortality. *JAMA Cardiology*.

[34] Dua S., Bhuker M., Sharma P., Dhall M., Kapoor S. (2014). Body mass index relates to blood pressure among adults. *North American Journal of Medical Sciences*.

[35] Gilbert-Diamond D., Baylin A., Mora-Plazas M., Villamor E. (2009). Correlates of obesity and body image in Colombian women. *Journal of Women’s Health*.

